# From infancy to toddlerhood: A 3D analysis of facial asymmetry in children with and without orofacial clefts

**DOI:** 10.1007/s00784-025-06484-1

**Published:** 2025-08-02

**Authors:** Katarína Martinková, Lenka Kožejová Jaklová, Karolina Kočandrlová, Jiří Borský, Ján Dupej, Alexander Morávek, Jana Velemínská

**Affiliations:** 1 Department of Anthropology and Human Genetics, Faculty of Science, Charles University, Viničná 7, Prague, 128 00 Czech Republic; 2 Department of Histology and Embryology, Third Faculty of Medicine, Charles University, Ruská 2411, 100 00 Prague, Czech Republic; 3 Department of Biology and Medical Genetics, Second Faculty of Medicine, Charles University and Motol University Hospital, V Úvalu 84, Prague, 150 06 Czech Republic; 4 Department of Otorhinolaryngology, Second Faculty of Medicine, Charles University and Motol University Hospital, V Úvalu 84, 150 00 Prague, Czech Republic; 5 Department of Anatomy, Faculty of Medicine in Hradec Králové, Charles University, Šimkova 870, 50003 Hradec Králové, Czech Republic

**Keywords:** Infancy & toddlerhood, 3D landmarks-based methods, Polygonal mesh analyses, Geometric morphometrics, Directional asymmetry, Facial development with cleft

## Abstract

**Objectives:**

This study investigates facial directional asymmetry (DA) in early childhood. Using 3D imaging, it aims to assess the DA progression in healthy controls and children with unilateral cleft lip (CL) and unilateral cleft lip and palate (UCLP) following early neonatal cheiloplasty and palatoplasty.

**Materials & methods:**

The sample consisted of 105 children (groups: cleft 42, control 63) aged 0.2–2 years. DA was analysed using geometric morphometrics, including 3D landmark-based and polygonal mesh analyses. Multivariate statistics were used for assessing DA significance and age group differences.

**Results:**

Controls showed no DA in landmarks and only mild protrusion of the right hemiface, increasing with age. In groups with cleft, DA was more pronounced in UCLP and especially in the middle of the face. While older UCLP children showed more asymmetrical faces, DA in children with CL became more comparable to that in controls with increasing age. Older children showed no statistical difference between control and CL in the landmark and polygonal maps parts.

**Conclusions:**

After surgical treatment, a DA pattern was identified, and it was specific for both cleft types and age categories. For both diagnoses, the most notable feature is the protrusion at the cleft site, likely related to post-surgical scarring, but in the UCLP group, it is also the hypoplastic nasal wing and a growth insufficiency of the cheek on the cleft side.

**Clinical relevance:**

3D methodologies provide insight into asymmetry progression and surgical outcomes, supporting improved cleft management for enhanced aesthetic and functional results.

## Introduction

Early childhood, particularly the first two years of life, is a critical period for craniofacial growth and development, marked by rapid morphological changes that lay the foundation for later facial structure and function [1]. During this time, both genetic factors and environmental influences interact to shape the face, and disruptions from congenital anomalies, such as orofacial clefts, can significantly alter typical growth patterns.

Orofacial clefts, including unilateral cleft lip (CL) and unilateral cleft lip and palate (UCLP), are associated with a range of functional and aesthetic challenges. Individuals with CL or UCLP often exhibit compromised orofacial functions, including swallowing, feeding, and speech, as well as impaired maxillofacial growth and noticeable facial asymmetry, particularly in the middle face, where the presence of a cleft causes growth restrictions [2–4]. Due to other negative aspects associated with the orofacial clefts, surgical intervention is recommended at an early stage. Initial lip repair (early cheiloplasty in this sample) is prioritized soon after birth, followed by palatoplasty in cases of complete clefts (UCLP). This treatment protocol is reserved for newborns in excellent health and requires an advanced interdisciplinary approach [5].

Facial asymmetry is regarded as an essential factor in evaluating the success of the surgical repair of orofacial clefts, particularly due to its strong association with psychosocial concerns such as perceived attractiveness and intelligence [6, 7]. Pronounced asymmetries in the middle face are often viewed as less attractive, which can impact social interactions and self-esteem [8, 9]. Post-operative evaluation of facial development in patients with cleft considers both sagittal and transverse facial dimensions, with surgical therapy aimed at reducing maxillary hypoplasia or retrognathia. In frontal view assessment, facial asymmetry remains a primary metric, as it is often one of the most visible indicators of disrupted facial growth patterns [3]. Facial asymmetry is especially evident in patients with complete clefts, stemming from underlying disruptions in midfacial tissue development, and is apparent both in untreated newborns and those who have undergone surgical intervention. In view of this, achieving symmetry close to typical facial morphology is a primary objective of surgical treatment.

Focusing on early childhood is particularly valuable for understanding the development of facial asymmetry because this period is marked by dynamic changes in facial proportions and structure. Given the lack of standardized methods for quantifying and comparing facial asymmetry in infants and young children, particularly within the context of orofacial clefts, research that captures this developmental window is crucial [10, 11]. In addition, evaluating and quantifying asymmetry is essential not only for assessing individual outcomes but also for comparing the effectiveness of different surgical techniques and their timings [12, 13]. Research into asymmetry further contributes to optimal final morphology of the face, which is influenced by the anomaly itself, individual growth potential, and the chosen surgical procedure [7, 14]. Surgical treatment leading to improved growth of the middle face and lip function should positively correlate with less asymmetry and more favourable outcomes regarding this aesthetically and psychosocially significant aspect [15].

The present study focuses on two main objectives, the achievement of which will help us answer our hypotheses: **(1)** To assess the degree of facial directional asymmetry (DA) in healthy children (non-cleft controls) and patients with CL and UCLP during the first two years of life, with particular focus on regions directly impacted by surgical intervention, including the temporal region, nasal and philtral area, buccal area and lips. We hypothesised that the level of asymmetry will vary depending on the type and extent of cleft, with more severe forms (UCLP) presenting higher degrees of asymmetry. Moreover, we expected that surgical repair will moderate the DA in certain regions, but a measurable discrepancy will persist compared to non-cleft controls, given the inherent disruptions in early facial development. **(2)** To describe differences and progression in manifestation of the shape DA in groups of patients with CL and UCLP at 0.2–0.5 years of age and at 1.6-2.0 years of age. Here we hypothesize that the DA will increase during the first two years, more significantly in both types of cleft defects, particularly in the UCLP group, where the face on the cleft side is typically hypoplastic.

Research into facial DA is a special kind of shape analysis. Therefore, a combination of 3D stereophotogrammetry, landmark-based, and polygonal mesh analyses was used to achieve the objectives and to test the hypotheses. This methodological framework enables a nuanced, multidimensional assessment of the DA, providing insights into the progression of craniofacial morphology in early childhood among patients with orofacial clefts.

## Materials & methods

### Ethical statement

The Institutional Review Board of Charles University, Faculty of Science, Prague, Czech Republic, approved this study with approval number 2024/11. All parents of children in the study provided signed, written informed consent for long-term research of their children.

### Study population

The study included a cross-sectional sample of 105 children aged 0.2–2.0 years. The total sample was divided into two groups, namely T0 (0.2 to 0.5 years) and T1 (1.6 to 2.0 years). These were patients with non-syndromic clefts (CL, UCLP) and healthy controls in the same age range. All patients with clefts were matched so that the cleft always occurred on the left side (if the cleft occurred on the right side the scan was flipped). The total number of subjects included in the study is summarized in Table 1.

**Table 1:** The total number of patients with clefts and controls used in the study (cross-sectional sample). T0 = children at 0.2–0.5 years, T1 = children at 1.6-2.0 years, cl = group with unilateral cleft lip, uclp = group with unilateral cleft lip and palate

	T0 (0.2–0.5 years)	T1 (1.6–2.0 years)	total
CL	11	10	21
UCLP	12	9	21
controls	31	32	63
total	54	51	**105**

The inclusion criteria for this study were defined to ensure a homogenous patient sample. Eligible participants were full term neonates (gestational age between 37 + 1 and 41 + 6 weeks) presenting with non-syndromic clefts (CL, UCLP). Additional requirements included uniform timing for cheiloplasty, performed within the first 14 days after birth (early neonatal cheiloplasty), with all surgeries conducted by the same surgeon to maintain procedural consistency. Only neonates in excellent health at birth, with stable circulatory and respiratory systems, were considered to allow for early surgical intervention. Exclusion criteria encompassed the presence of serious congenital developmental defects (e.g., heart, central nervous system, kidneys, gastrointestinal tract) and any severe pathologies such as infections, coagulation disorders, anaemia, cardiac arrhythmias, etc. Patients within our laboratory’s database who did not meet these criteria were excluded from the study.

These criteria were similar for the control group, which consisted of age-matched healthy newborns and toddlers. Children included in the study were full term (37 + 1 to 41 + 6 weeks of gestation), without craniofacial deformities, without any plastic surgery, and without impaired facial muscle function.

All of the children (controls and patients with clefts) participating in the study were of Czech nationality.

### Surgical therapy

All children with orofacial cleft underwent early neonatal cheiloplasty at a mean age of 6 ± 4 days after birth to repair the lip, which was supplemented by palatoplasty at a mean age of 0.9 ± 0.3 years in patients with UCLP. In the UCLP group, the division of the study subjects into age groups therefore reflected operations performed according to the Czech surgical protocol in early childhood, with the T0 group corresponding to the pre-palatoplasty condition, while the T1 group included patients who had already undergone palatoplasty. This allowed us to observe the development of facial symmetry concerning surgical interventions. In other words, the T0 group in both CL and UCLP patients followed the development of facial symmetry affected only by early neonatal cheiloplasty, whereas the T1 group in UCLP patients followed the combined effect of cheiloplasty and palatoplasty on the development of facial symmetry.

As far as cheiloplasty is concerned, all patients were treated using the modified Tennison method. Compared to the original Tennison method, this modification involved two mucosal flaps obtained by repositioning the cleft margins. The flaps were used to deepen the atrium and floor of the nasal cavity and the upper oral vestibule. In the final stage of the operation, the alveolar cleft was closed using soft tissue. The surgical procedure itself was supplemented with high-frequency tympanometry and determination of otoacoustic emissions. If a middle ear secretion was detected, a spot tympanotomy was performed, and the secretion was removed to prevent irreversible changes in the middle ear cavity [16]. The next step in the surgical protocol was obtaining dental cast impressions taken by a plastic surgeon.

The total duration of surgery varied depending on the severity and extent of the cleft but usually lasted 60 to 120 min. After the lip surgery, patients were transferred to the neonatal intensive care unit for further care, where they remained for an average of 3 days. It should be kept in mind that a prerequisite for successful early lip repair is the provision of specialized technical equipment, trained personnel, and a multidisciplinary team of specialists involved in surgical care. Approximately 10 months after cheiloplasty, patients with UCLP underwent a Furlow double opposing Z-plasty [5].

### Data collection process

All subjects were scanned using a 3D 3dMDface System optical scanner (3dMD Inc., Atlanta, GA, USA) and Vectra 3D scanner (Canfield Scientific, Inc., Fairfield, NJ, USA). Both 3D scanners used are safe and apply non-invasive methods for facial imaging based on the optical scanning principle (stereophotogrammetry). The scanning was performed using a synchronised dual-camera system consisting of six machine vision cameras and an industrial flash system. The capture speed is approximately 1.5 milliseconds. At the end of the scanning process, the 3D polygonal meshes (x, y, z Cartesian coordinate system) with colour texture are rendered in 7 s. The mesh accuracy was established at < 0.2 millimetres or better. The scanned field of view was established on a 180-degree capture, which represents ear-to-ear capture. Children were scanned by two experienced researchers in neutral facial expression, without significant facial hair and any involuntary movements. In case of any movements or changing from a neutral expression to another, individuals were scanned again. The final high-resolution surface model was created using the associated Mirror PhotoTools or 3dMD software.

### Data editing and landmarking

The 3D polygonal meshes were processed using Rapidform 2006 software [17]. Mesh postprocessing consisted of hole and mesh error removal, as well as the removal of excess facial parts (hair, ears, lower part of the chin, and neck area), and mesh decimation to approximately 25k vertices. All 3D scans were unified to a single orientation. This step ensures consistency in visualization and subsequent asymmetry analysis.

MorphoJ software for 3D landmark analysis [18] and Morphome3cs software for polygonal mesh analysis [19] were used for further steps. Landmark and surface analyses are independent but complementary. Each represents a different type of methodological approach and final results. The 3D landmark analysis is less detailed and allows DA manifestations to be shown in right-left shifts and cranio-caudal shifts, but only in areas where landmarks are placed. Polygonal mesh analysis is a very detailed method where protrusive and retrusive areas on the whole surface are visible. Using these two methods together enables a description of asymmetrical shifts in any direction.

**Fig. 1:**
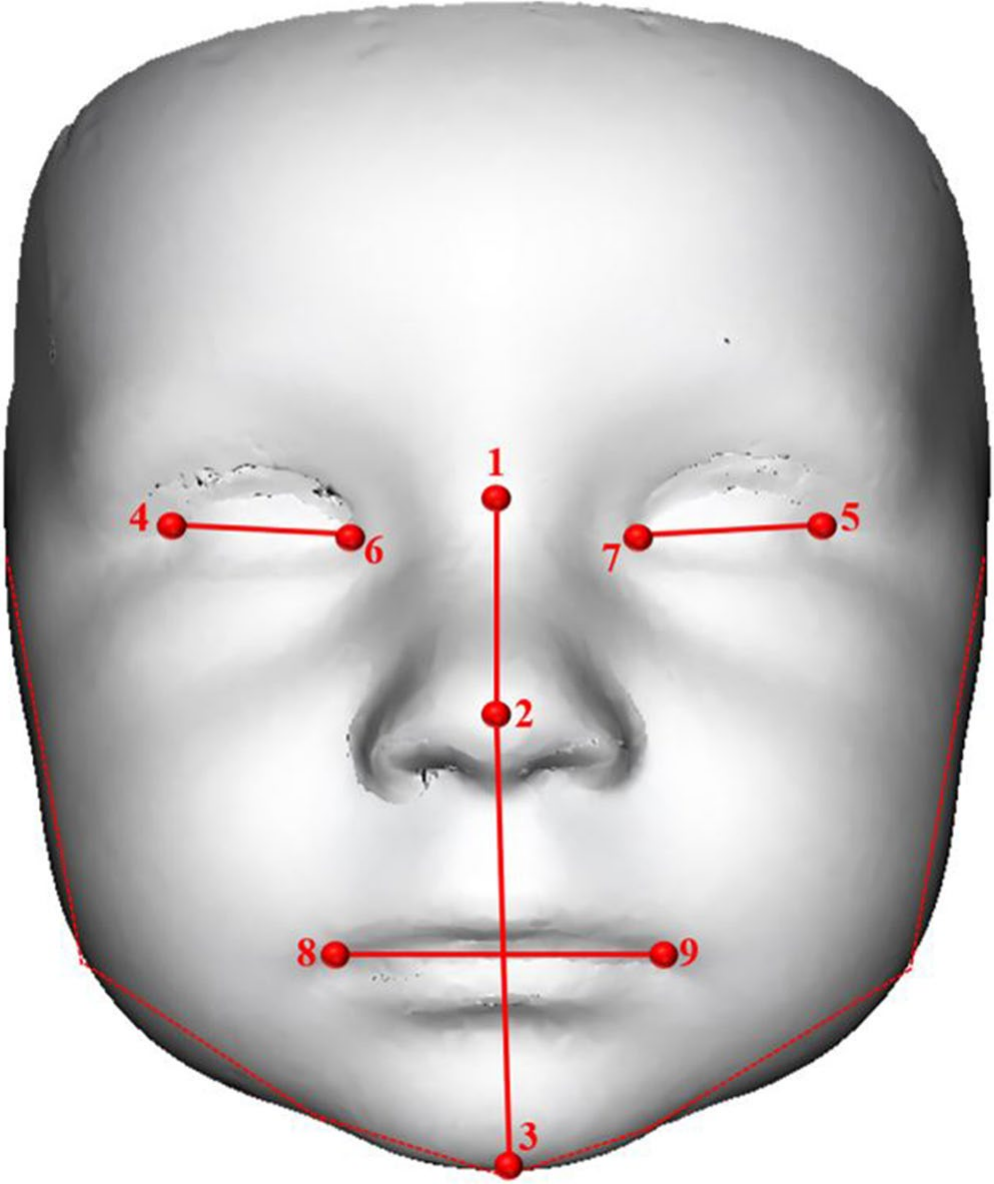
Placement of nine 3D landmarks on an average face. Unpaired landmarks are: 1 – *nasion*, 2 – *pronasale* and 3 – *gnathion*. Paired landmarks (one copy on the right side and one copy on the left side) are: 4, 5 – *exocanthion*, 6,7 – *endocanthion* and 8, 9 – *cheilion*

The landmark digitization was carried out in Morphome3cs software (http://www.morphome3cs.com) [19]. Nine different 3D landmarks (Fig. 1) were digitized by one technical operator (to avoid interobserver error). All landmarks were represented by the Cartesian coordinates *x*,* y*,* and z*. Digitized landmarks included unpaired landmarks placed in the midline of the face (*nasion*,* pronasale*,* gnathion*) as well as paired landmarks, always one copy on the left side and the second copy on the right side (*exocanthion*,* endocanthion*,* cheilion*). The Cartesian coordinates of these nine landmarks were also exported and used for the 3D landmark-based method separately from mesh analyses.

### 3D landmarks-based analyses

Digitized 3D landmarks were first analysed using geometric morphometrics alone. For landmark analysis in MorphoJ software [20] the object asymmetry approach was applied, which allows description of right-left and cranio-caudal shifts. This kind of approach represents a human face (or any other object) that is symmetrical in itself, in the sense of two mirrored halves with a symmetrical axis. The object is specified by two landmark configurations: paired landmarks with one copy of the configuration on the right and left halves of the object, and then with the configuration of unpaired median landmarks [18, 21]. This method is based on the original landmark configuration and relabelling them into a mirrored configuration of landmarks. These two configurations then create a new, symmetric configuration together, which allows comparison of the new mirrored configuration with the original shape, or comparison of an ideal symmetrical configuration and the original configuration. After comparison, the asymmetrical component of shape asymmetry (the difference between the original and ideal symmetrical landmark configurations) is created [18].

After GPA, the Cartesian coordinates were recalculated into symmetrical and asymmetrical components, while the asymmetrical one was used in the following 3D landmark analyses. Each group of individuals (control, CL, UCLP) was assessed separately at both time points. Firstly, the absence/presence and visual manifestation of DA in groups was assessed by Procrustes analysis of variance using default parameters (Procrustes ANOVA). We tested 6 different groups, which means 6 different Procrustes ANOVAs (at T0 time: controls, CL, UCLP and at T1 time: controls, CL and UCLP).

Since the asymmetrical component was widely multidimensional, for a reduction of these data dimensions Principal Component Analysis (PCA) was performed. Further statistical analyses used principal components (PCs), which cumulatively explained at least 75% (the first 5 PCs) of overall variability.

Canonical Variate Analysis (CVA) was used to test differences between the average DA shape of the control, CL, and UCLP groups at different times (T0 and T1).

The level of statistical significance was set at 5% for most of the tests. For Procrustes ANOVA, a Bonferroni correction [22] was applied because of using 6 different tests. The statistical significance level was corrected at 0.0083.

### Polygonal mesh analyses

After landmark digitization, analyses of 3D polygonal meshes were carried out using Morphome3cs software (http://www.morphome3cs.com/) [19].

As the 3D models did not demonstrate vertex homology even after editing, the first step was to achieve homology representing the vertices with the same index defining the same anatomical characteristics. The initial phase of this process is based on the application of landmarks to the 3D models. Landmarks were used for a rigid pre-alignment of the models with a selected random base facial mesh as a template for alignment. Base mesh vertices were transferred to all meshes automatically. Registration of base mesh onto dataset meshes was processed by means of the fully automatic non-rigid registration Coherent Point Drift (CPD) algorithm [23]. Vertex homology was established using Coherent Point Drift-Dense Correspondence Analysis (CPD-DCA) [24] and was allowed by the projection of deformed base mesh vertices to the closest points on all other meshes. Vertices without appropriate matching were excluded from the analysis. When CPD-DCA analysis is performed, all vertices can be assessed as 3D landmarks (known as quasi-landmarks, ordinary landmarks).

Prior to data evaluation, our measurement error was calculated to be 0.25 mm [25]. The final alignment of meshes was provided by Generalized Procrustes Analysis (GPA). The process of achieving shape directional asymmetry (DA) of the face comprises using a mirror copy of all meshes from the dataset and registering them onto their original non-mirrored copies. Mesh mirroring was an automatic process represented by adding an opposite spatial position to paired mesh landmarks. This means that unpaired landmarks were unaffected by mirroring while paired landmarks swapped their positions with each other [26].

Visualization of the facial shape DA on 3D polygonal meshes was provided by colour-coded maps. There were colour-coded maps for each group (average shape asymmetry) and also maps illustrating the differences in average asymmetry between the younger and older age groups. Red shaded colours indicate protrusion/ventral shifts of the facial areas, while dark red indicates the most prominent protrusion. Blue shaded colours indicate the retrusion/dorsal shifts of the facial area, and dark blue represents the most retrusive parts. Green shades indicate neutral areas without protrusion or retrusion, or show group shape differences (for difference visualisation, the group superimposition was used). In maps with superimposed groups, there was always a comparison between the second visualised group and the first group. This means that during the interpretation of superimposed maps the younger children (T0) – first group were described contrary to the older children (T1) – second group.

After polygonal mesh analysis, the PCA score (from meshes) was exported. This PCA score was used to describe variability across the dataset and for assessing differences between groups of children (control, CL and UCLP) in T0 and T1. All PCA analyses were performed in R software [27] and some steps were performed using specialized packages (one of them was used for reading.csv files [28]).

Firstly, assumptions for parametric testing were verified by: Box´s M [29] test and Levene tests [30]. These were not violated. Box´s M test for T0 was p-val = 0.1511 and for T1 it was p-val = 0.3509. The Levene test showed in T0 p-val: PC1 = 0.7060, PC2 = 0.9005, PC3 = 0.3819 and PC4 = 0,0631. In T1 it showed p-val: PC1 = 0.0573, PC2 = 0.2516, PC3 = 0.8458 and PC4 = 0,4735.

The differences in average shape asymmetry between groups (children with/out cleft in T0 and T1) represented by PCA scores of the first 4 PCs (based on the broken stick method) were statistically tested by MANOVA [31] and post-hoc Hotelling’s T² tests [32]. The adjusted p value for post-hoc tests was set at 0.0167 by the Bonferroni method (3 tests). For visualisation of group differences Canonical Variate Analysis (CVA) was used [33].

To assess the statistical power of our analyses, we performed a post-hoc power analysis using combined PCA data (PC1–PC4) from both T0 and T1 groups. A permutational RRPP model [34] was used, with factors including cleft type (CL, UCLP, control) and measurement time (without interaction). Effect size (Cohen’s f) was calculated from the resulting F-statistic (F = 3.1709; df₁ = 2, df₂ = 96), yielding Cohen’s f = 0.2570. With actual combined group sizes of CL = 21, UCLP = 21, and control = 63, the effective harmonic mean sample size per group was 27.00. The achieved statistical power at α = 0.05 was 0.516, indicating that approximately 50 subjects per group would be required to reach the conventionally recommended power of 0.8.

## Results

### Result of directional asymmetry in 3D landmarks analysis

#### DA manifestation in 3D landmarks analysis

In the first step of facial DA evaluation, landmark asymmetry analysis was a applied – a simple method showing right-left and cranio-caudal directions.

Our results showed that there is significant facial DA in the following groups (Table 2): controls (at T0 time), UCLP (at T0 and also T1 times). The individual factor contributing to final shape and asymmetry was significant in every tested group.

**Table 2:** Procrustes ANOVA results. Significance level was settled after bonferroni correction at 0.0083. T0 = children at 0.2–0.5 years, T1 = children at 1.6-2.0 years. DA = facial directional asymmetry, cl = group with unilateral cleft lip, uclp = group with unilateral cleft lip and palate

***P*** **val**	**CONTROLS**			
	**T0**		**T1**	
	**Individual**	**DA**	**Individual**	**DA**
p	< 0.0001	**0.0025**	< 0.0001	0.2734
	***CL***			
	**T0**		**T1**	
	**Individual**	**DA**	**Individual**	**DA**
p	< 0.0001	0.0480	< 0.0001	0.2284
	***UCLP***			
	**T0**		**T1**	
	**Individual**	**DA**	**Individual**	**DA**
**p**	< 0.0001	**0.0037**	< 0.0001	**0.0025**

**Fig. 2:**
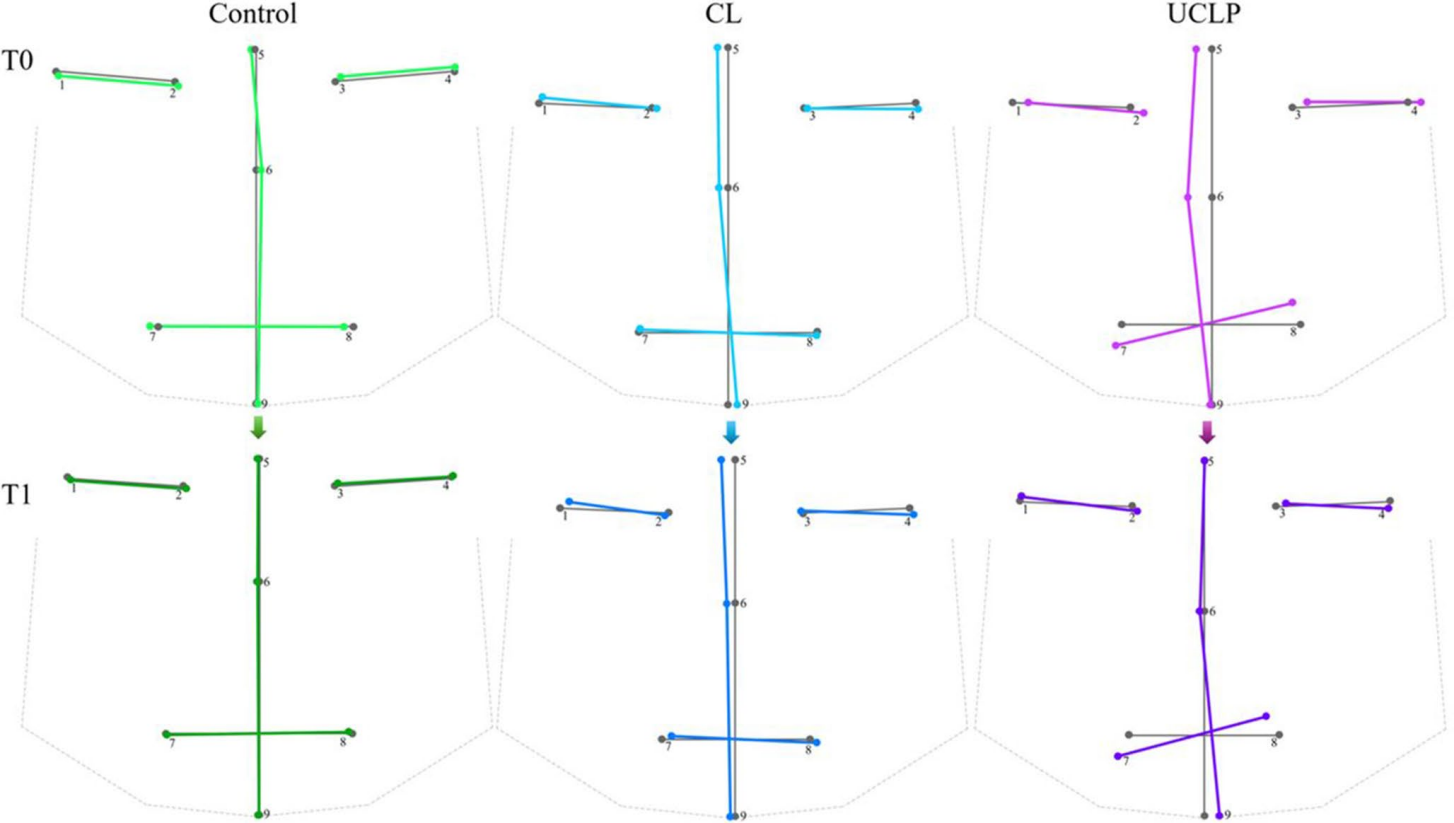
Mean facial DA from the frontal view visualised by wireframe graphs. The grey wireframe graph always shows the mean symmetrical shape for each group. Coloured wireframe graphs are for comparison of facial DA to ideally symmetrical shapes. The light green shows mean DA for control children at time T0 (0.2–0.5 years) and the dark green shows mean DA for controls at time T1 (1.6-2.0 years). The light blue shows mean DA for the CL group at time T0 (0.2–0.5 years) and the dark blue shows mean DA for CL patients at time T1 (1.6-2.0 years). The light purple shows mean DA for UCLP patients at time T0 (0.2–0.5 years) and the dark purple shows mean DA for UCLP individuals at time T1 (1.6-2.0 years). Numbers 1 to 9 represent 3D landmark numbers. A scale factor of 10 was applied to enhance visualisation of facial asymmetry (default scale factor was 1). CL = group with unilateral cleft lip, UCLP = group with unilateral cleft lip and palate

The manifestation of facial DA is shown in Fig. 2 In the control group, facial DA manifested only slightly. Younger children showed a lower position of the right eye compared to the left eye. In midline landmarks, there were deviations, first to the right side – *nasion* and secondly to the left side – *pronasale*. Older children from the control group were very symmetrical, almost without any deviations from the symmetrical shape mean. Younger children in the CL group showed a wider DA asymmetry than the controls. This asymmetry was mainly manifest in the midline area, with right-sided shifts of the *nasion* and *pronasale* and a left-sided shift of the *gnathion* from the symmetrical midline. The were also small DA deviations of the eyes and the mouth, these structures showing a slight clockwise rotation. Older children with CL showed a similar facial DA trend to younger children with the same type of cleft, but the landmarks of the midline were shifted only to the right side (*nasion* the most) of the symmetrical midline. The group of younger children with UCLP showed wider DA manifestation. There was very visible right-sided deviation of the *nasion* and *pronasale* from the midline. The right mouth corner was lower than the left corner, and in general, the mouth line deviated strongly from the symmetrical mean. The last group, older children with UCLP after palatoplasty, showed DA as well; however, the manifestation of this asymmetry was less visible than in the younger children. The facial midline was more symmetrical except for the left-shifted *gnathion*. Asymmetry of the mouth was similar to the younger group. In general, we observed a lesser manifestation of facial DA in children in the older age group and in the case of UCLP after palatoplasty.

#### DA variability description in 3D landmarks analysis

Principal Component analysis (PCA) for each tested group at T0 showed that the first Principal Component (PC) represents more than 34% of the shape variability and the second PC represents more than 20% of the overall variability. The fist 4 PCs represent more than 75% of the cumulative shape variability, and 9 PCs explain 100% of the variability.

PCA of children at T1 showed that the first PC represents more than 25% of the overall variability and the second PC more than 18%. Cumulatively, the first 5 PCs show more than 75% of the overall variability, 9 PCs explaining 100% of the variability.

#### DA differences between groups in 3D landmarks analysis

Canonical Variate Analysis (CVA) showed significant differences between the tested groups at T0 and T1 (Table 3). Groups at T0 were significantly different from each other (based on Procrustes distances). The DA in T1 groups was significantly different between groups, except for the control and CL groups.

**Table 3:** Results of CVA for facial DA in each group at T0 (0.2–0.5 years) and T1 (1.6-2.0 years, in patients with UCLP after palatoplasty). Results are presented as procrustes distances and their p-values. CL = group with unilateral cleft lip, uclp = group with unilateral cleft lip and palate

	T0–0.2–0.5 years of age			T1–1.6-2.0 years of age		
Procrustes distances		*CL*	*UCLP*		*CL*	*UCLP*
	*UCLP*	0.0191		*UCLP*	0.0215	
	*Control*	0.0128	0.0207	*Control*	0.0111	0.0147
p value		*CL*	*UCLP*		*CL*	*UCLP*
	*UCLP*	0.0491		*UCLP*	0.0162	
	*Control*	0.0219	0.0207	*Control*	0.0716	0.0023

**Fig. 3:**
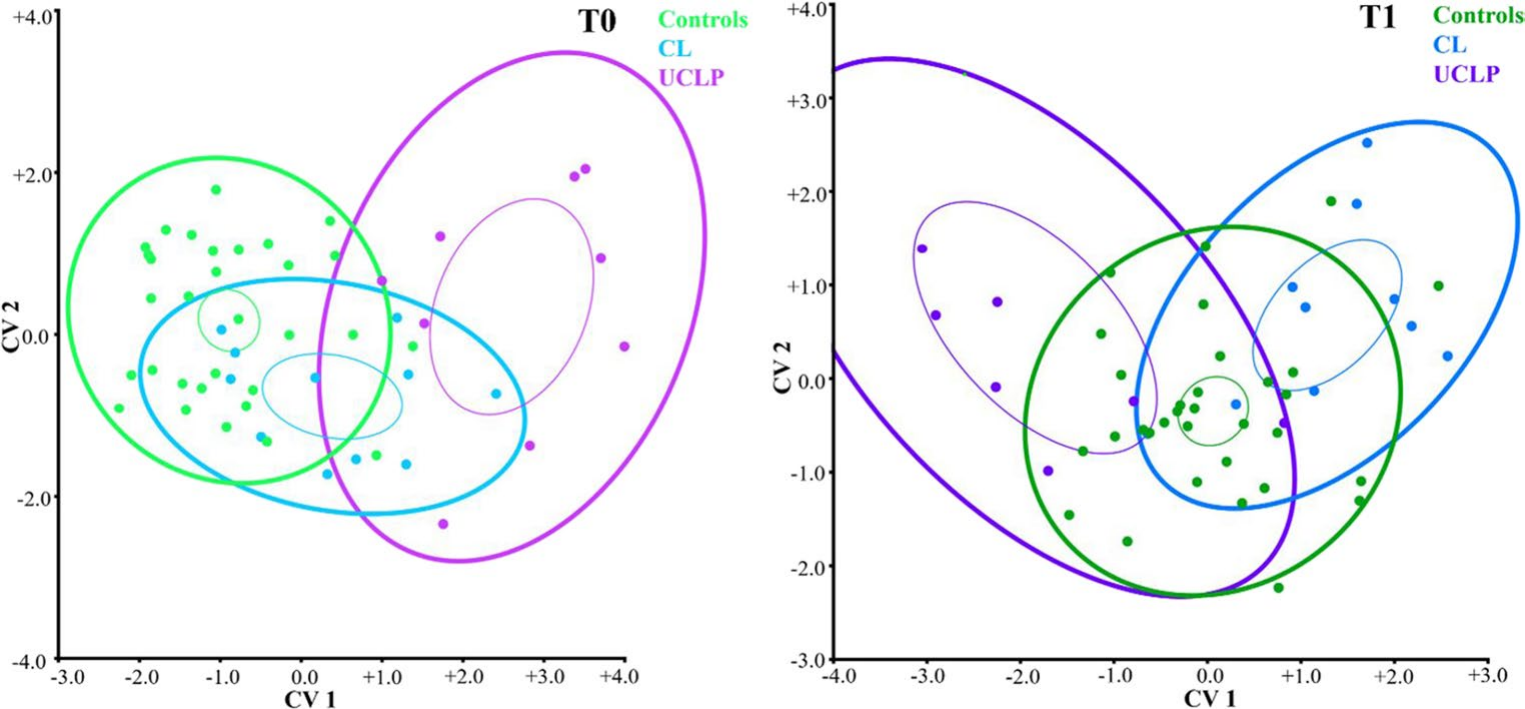
A CVA scatter plot visualizing the shape DA variability for the 3D landmarks used. This plot visualises the space defined by Canonical Variates (CV) 1 and 2 for the control group and CL and UCLP groups at T0 (0.2–0.5 years, group with UCLP before palatoplasty) and at T1 (1.6-2.0 years, group with UCLP after palatoplasty). The bigger and thicker 95% confidence ellipses represent variability in all three groups separately. Smaller and thinner ellipses represent the DA shape means of each group. CL = group with unilateral cleft lip, UCLP = group with unilateral cleft lip and palate.

Figure 3 provides a visualization of CVA results for the T0 and T1 groups. The control and UCLP groups at T0 were the most distant from each other, and the CL group between them. However, at T1, the control group was in the middle of the variability spectrum and the CL group seemed to be closer to controls. Also, after surgery, the UCLP group moved closer to the controls, but still with relatively wide variability

### Result of directional asymmetry in polygonal mesh analysis

#### DA manifestation in polygonal mesh analysis

**Fig. 4:**
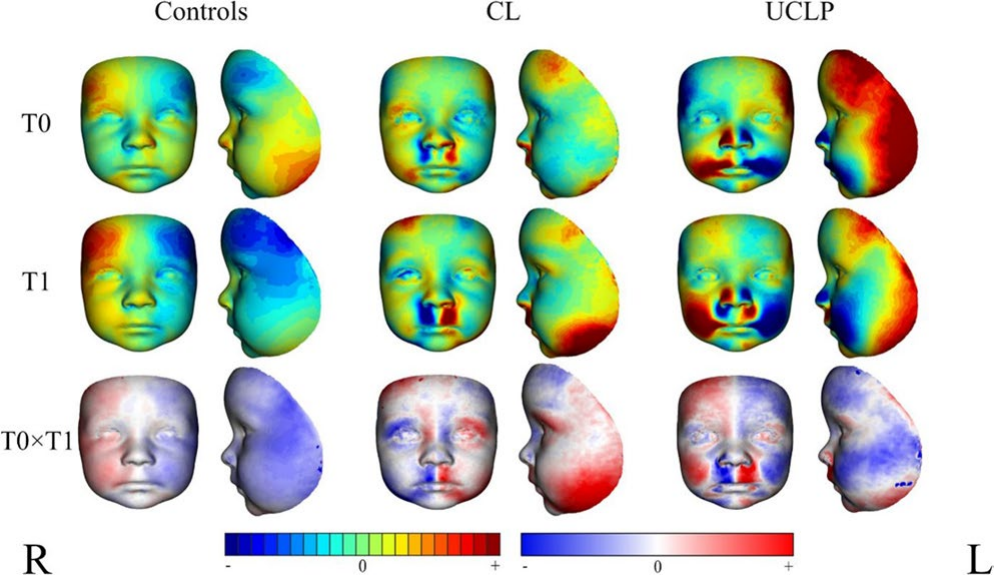
Colour-coded maps visualising age-related changes of facial shape DA between the youngest (T0 at 0.2–0.5 years) and oldest (T1 at 1.6-2.0 years) age categories, in groups with cleft (CL = group with unilateral cleft lip, UCLP = group with unilateral cleft lip and palate) and controls. Red-coloured shades indicate positive values of anterio-posterior DA (protrusion), while inverted, blue-coloured shades depict negative values of DA (retrusion). To compare older toddlers with younger infants, a blue-red-white scale was used, with red indicating areas of positive values of DA (protrusion), while blue represented negative values of DA (retrusion). R – right side (opposite side from the cleft), L – left (cleft side)

Colour-coded maps (Fig. 4) illustrate significant changes in DA across various facial regions among the different groups (CL, UCLP, controls), as a function of age (from the youngest to the oldest age category). This analysis focuses on the development of DA across the study period, stratified by specific facial regions: the upper face (forehead and temporal region), middle face (nose, philtrum, mouth and cleft area), and lower face (chin).

Within the upper face, DA was absent in the central region of the forehead; however, distinct asymmetrical areas were observed in the temporal region, with trends differing markedly between cleft-affected patients and controls. Among patients with clefts, the temporal region exhibited protrusion on the cleft side (left), contrasting with retrusion on the opposite side. This pattern was particularly pronounced in patients with UCLP, reflecting the increased severity of the cleft. In contrast, healthy individuals under two years of age demonstrated a protrusive left temporal region with corresponding retrusion on the right. Comparison of the youngest and oldest age groups (0.2–0.5 years and 1.6-2.0 years) confirmed that the most pronounced DA was evident in the most severely affected group (UCLP) in the temporal region, with the oldest group having a more retrusive cleft side compared to the newborns. Compared to the youngest individuals with CL, older individuals suffered from a striking asymmetry in the supraorbital arches, with a more protrusive side of the cleft. However, this trend was consistent with the other groups evaluated, including controls, and thus followed the typical DA located in the temporal region even in the healthy population.

The middle part of the face, as anticipated, showed the greatest degree of cleft-related asymmetry. Controls exhibited a relatively symmetrical nasal region, similar to those with CL, apart from a slight protrusion on the right side of the nose in the CL group. In patients with UCLP, however, nasal asymmetry was substantial, characterized by persistent protrusion on the side opposite the cleft (right) across all time points, without evidence of improvement over the study period. The philtrum appeared even more asymmetrical in patients with both types of clefts, with persistent protrusion on the cleft side (left). This asymmetry was limited to the philtrum in individuals with CL; however, in the UCLP group, the asymmetry extended to adjacent cheek areas, displaying notable retrusion on the cleft side and corresponding retrusion on the opposite side.

Comparison of the two age groups confirmed that the condition of the older individuals, regardless of cleft type, did not improve much, and that DA in the middle face remained significant, mainly at the point of cleft left where scar tissue was protrusive. The DA resulting from the presence of cleft was also reflected in the cheek area in 1.6-2.0 years toddlers, with the cheeks on the CL side of the cleft being more protrusive, whereas in the UCLP patients, only the scar was protrusive, and the cheeks on the cleft side showed retrusion.

Surprisingly, the mouth region, particularly the lower lip, remained largely symmetrical across groups, especially in healthy controls and those with CL. Finally, the chin region in the lower face displayed symmetry in both control subjects and patients with CL. A mild protrusion was present on the cleft side in the UCLP group.

#### DA variability description in polygonal mesh analysis

The PCA showed for each tested group that the first PC represents more than 38% of shape variability. The second PC represents more than 26% of the overall variability. The first 4 PCs represent more than 75% of the cumulative variability (according to the broken stick method, the first 4 PCs were also used for the following analyses), while 20 PCs explained 100% of the variability.

#### DA differences between groups in polygonal mesh analysis

The results of the MANOVA (for T0 and T1 separately) showed differences only at T0 (p value = 8.835e-07), while at T1 there were no differences between groups (p value = 0.3509). Post-hoc Hotelling´s T^2^ tests showed that at T0 there were statistically significant differences in directional asymmetry between: control children and children with CL (p value = 0.0001042), and control children and children with UCLP (p value = 3.601e-19). There was no significant difference in DA between children with CL and UCLP (p value = 0.1275).

**Fig. 5:**
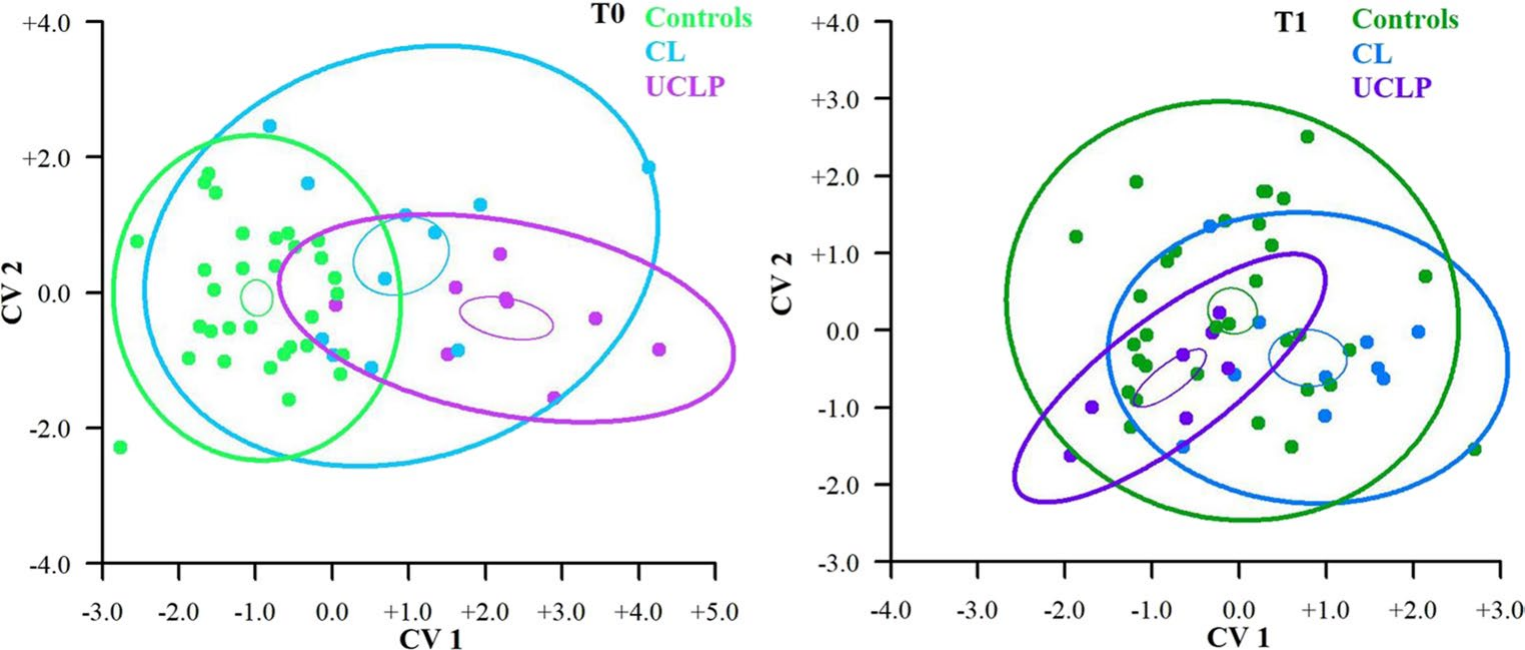
A CVA scatter plot visualizing the shape DA variability for PCA score extracted from polygonal 3D meshes. This plot visualises space of the CV 1 and CV 2 for: the control group and groups with CL and UCLP at T0 (0.2–0.5 years of age, group with UCLP before palatoplasty) and at T1 (1.6-2.0 years of age, group with UCLP after palatoplasty). The bigger 95% confidence ellipses represent variability in all three groups separately. Smaller ellipses represent the estimates of the DA shape mean of each group. CL = group with unilateral cleft lip, UCLP = group with unilateral cleft lip and palate

Visualization of differences in DA between groups of children was performed by CVA for T0 and T1 separately (Fig. 5). At T0, the control (the densest group) and UCLP groups were the most distant from each other, while the CL group was positioned between them. This trend is very similar to the CVA analysis based on 3D landmarks. At T1, the control group was mostly in the middle of asymmetry variability, and the groups of children with CL and UCLP were closer to the control group. Ellipses show that children with UCLP manifested the smallest variance of DA variability at T0 and especially at T1.

## Discussion

Our results provide a comprehensive view of facial shape directional asymmetry in infants and two-year-old children following early neonatal cheiloplasty (all patients) and, in cases with complete cleft (children with UCLP), palatoplasty. These findings offer a unique perspective on DA not only from a growth-related research standpoint but also in terms of methodological evaluation using geometric morphometrics. New findings on early DA progression reveal distinct patterns of asymmetry in different facial regions. By employing advanced 3D imaging techniques, this study not only highlights differences in facial morphology between affected and unaffected groups but also underscores the influence of early surgical interventions on craniofacial development.

### Methods

From a methodological perspective, we used two different techniques that are innovative and well-established in addressing directional asymmetry [35, 36]. The combined use of 3D landmark and polygonal mesh analyses allows for a multi-dimensional assessment of the DA across all spatial axes (x, y, and z), providing detailed information on asymmetrical shifts in the left-right, cranio-caudal, and antero-posterior directions. This dual approach enhances accuracy in describing asymmetry patterns, capturing subtle shifts that may not be observable through traditional methods focused solely on anatomical landmarks or single-plane analysis [18]. This combined approach is particularly useful in clinical applications where precise quantification of the DA is crucial for tracking post-operative outcomes and informing treatment adjustments [37]. The current study’s results are robust and provide an in-depth view of facial asymmetry changes over 2 years in early childhood.

### Surgical approach

Early neonatal cheiloplasty, performed within the first two weeks of life, is becoming a relatively more commonly used surgical approach for managing orofacial clefts due to current advanced medical methods [38, 39]. This early intervention aims to restore anatomical continuity and support more symmetrical craniofacial development during childhood. Early neonatal repair aligns with current understandings of craniofacial plasticity [40], suggesting that intervention during a time of rapid growth may improve aesthetic and functional outcomes by supporting symmetrical development of soft and hard tissues around the point of cleft.

Several studies have confirmed that cheiloplasty (lip repair), without considering its timing, positively influences facial symmetry by promoting balanced growth across the middle face and reducing the probability of compensatory asymmetrical development [7, 41, 42]. Specifically, lip repair seems to facilitate soft tissue alignment in children with clefts, which may contribute to improved symmetry of the nose, philtrum, and cheeks [41, 42]. However, according to Al-Rudainy et al. [41], symmetry scores after lip surgery did not change in the lip and chin regions. Complete symmetry remains challenging to achieve, especially in patients with a complete cleft with severe tissue deficits.

The modified Tennison technique, as applied by Borský et al. [5], involves individual design and reorientation of muscles, which may offer better results in nasolabial symmetry compared to the classic Tennison method. The modification of this method allows better three-dimensional reconstruction of muscles and formation of the philtral column, which may potentially reduce postoperative asymmetry. The classic Tennison technique can lead to less than optimal muscle continuity, which may contribute to residual asymmetry of the orbicularis oris function and scar contracture patterns [43, 44].Palatoplasty, as well as subsequent surgical interventions on facial morphology, can significantly impact facial symmetry [45]. Studies indicate a potential trade-off with palatoplasty: while it effectively reduces cleft-related asymmetries, it may also limit maxillary growth in the sagittal direction, potentially resulting in retrusion [14, 46]. Ross (1987) highlighted that palatoplasty can lead to long-term growth restrictions if performed too early. Similarly, Ousterhout et al. [46] demonstrated that early palatal surgeries, while beneficial for alignment, may restrict forward maxillary growth, contributing to retrusion in later developmental stages. Recent work by Hermann et al. [47] supports these findings, noting that palatoplasty aimed at achieving symmetry can create challenges in maintaining balanced maxillary projection over time.

In the case of palatoplasty, the Furlow double reverse Z-plasty used in the cohort of patients with UCLP aims to lengthen the soft palate and more physiologically reconstruct the *levator veli palatini* suspension, which has a positive effect on speech outcomes and palatal contour. However, this method may cause asymmetrical scarring or tension vectors due to the nature of the double opposing flaps, especially in patients with wider clefts, which could affect the growth patterns of the maxilla and palate [48].

Early neonatal cheiloplasty, despite its benefits, often does not fully correct facial asymmetries, especially in the middle face and nasolabial regions. Studies indicate that asymmetries in these areas can persist post-surgery, particularly in cases of UCLP, where residual imbalances may necessitate secondary interventions or orthodontic treatments [49–51]. Advances in surgical techniques aim to improve initial outcomes, but complete correction of midfacial asymmetry often remains challenging, requiring additional treatment as patients grow [52].

### 3D landmarks

The results focused on 3D landmark analysis showed that directional asymmetry is present in groups of newborn controls, newborns with UCLP, and two-year-olds with UCLP after palatoplasty. No asymmetry was detected at the 3D landmark level in any group of CL children, but this may result from the small sample size (because the manifestation of DA is quite pronounced). According to a study by Stellwagen et al. [53], it can be assumed that asymmetry is present in many individuals from an early age, even without facial pathology. The absence of detectable directional asymmetry in the CL group at both time points may be due to the dataset size (p-value 0.0480) and the significance level being adjusted to 0.0083. In healthy controls, asymmetry became statistically insignificant with age. However, in the UCLP group, asymmetry was statistically significant at both time points.

A study by Launonen et al. [54] has demonstrated that asymmetry decreases with age in healthy children across all facial regions. This trend in asymmetry, assessed through landmark analysis, was also observed in our study and was statistically significant. Among healthy children, asymmetry showed a slight shift toward the symmetrical average, although this shift was not very pronounced, as asymmetry itself was not markedly evident in newborns. This result partially aligns with a study by Hood et al. [7], where the authors reported no change in asymmetry with age in healthy children; however, as the authors note, they assessed an asymmetry score for the entire face, which may have missed subtle asymmetrical nuances. In the Launonen et al. [54] study, the primary asymmetry trend observed was a leftward deviation of the midline in the upper half, and to the right in the lower half, of the face. In our study, healthy newborns showed an opposite trend in the midline, and among toddlers, midline asymmetry was almost negligible.

In children with CL or UCLP, directional asymmetry was found to be more pronounced in these two groups than in healthy controls (independently of age or asymmetry assessment method), which is in agreement with several studies [7, 55–58]. Additionally, the UCLP group exhibited a higher degree of directional asymmetry than the CL group. These findings are consistent with those of Hood et al. [7]. It was also found in our results that the most significant change in asymmetry occurred in the nasal area of the UCLP group after palatoplasty, while the change was represented by symmetrizing of the nose area. Hood et al. [7] also noted that the greatest changes with age occurred in the nasal region for children with UCLP, while the mouth area became more symmetrical gradually and to a lesser extent, which also aligns with our results. Our older (T1) children with CL showed a tendency towards slight symmetrizing of the nose and chin area. This shift closer to the symmetrical mean was small, however, and landmark asymmetry in this group was only moderately pronounced. Older children after palatoplasty showed a significant reduction in asymmetry, particularly in the nasal area (at the points *nasion* and *pronasale*). The eye region became somewhat more symmetrical, though to a lesser extent. The mouth area remained equally asymmetrical, and the chin shifted to the left compared to its position before palatoplasty. Even though there is a relatively large variation between the methods used for asymmetry assessment, there is a general agreement that the biggest variability occurs in the nasal and/or upper lip area [7, 41]. As also stated by Hood et al. [7], significant asymmetry persisted in the mouth area even after surgical treatment. Although Al-Rudainy et al. [41] mentioned that surgery did not alter the magnitude of asymmetry score in the areas of the lower lip and chin, we found (on 3D landmarks) that the chin was more asymmetrical after palatoplasty. Furthermore, Bell et al. [12] add that, despite surgical treatment, asymmetry in cleft patients remains significant even at 10 years of age.

### Polygonal mesh analysis

As also reflected in the existing literature, the greatest degree of facial DA was observed in our sample in patients with UCLP, regardless of age category [7, 12, 59]. Persistent DA was revealed across the facial regions in cleft-affected individuals with UCLP, supporting the existing literature on the complex challenges of achieving facial symmetry in these cases [12, 55, 60]. Several studies have shown that although a well-chosen combination of surgeries has the potential to correct some manifestations of facial DA, significant imbalances often persist in areas such as the middle face and nasolabial region due to innate structural deficits associated with UCLP, even at later ages [41].

According to our results, the most prominent DA was seen in the nasolabial region at the point of cleft in patients with UCLP, and in patients with CL in both age categories. Persistent protrusion on the side of the cleft was observed in the 2-year-old patients, which is consistent with a longitudinal study by Hermann et al. [47], where it was shown that mandibular growth is often limited due to scarring and altered bone morphology after surgery. This limitation tends to accentuate asymmetry over time, especially in areas such as the nose and philtrum, similar as in our findings. Other studies indicate that facial asymmetry in patients with clefts tends to diminish with age, with children aged 8 to 10 years beginning to exhibit facial symmetry similar to that of their healthy peers [55, 59]. In our study, we observed an increase in asymmetric deviations in the philtrum and cheek areas. However, the CVA analysis indicated that overall asymmetry patterns are more similar to each other in older children (2 years of age), especially the control and CL groups.

Al-Rudainy et al. [41] found that lip correction using the modified Millard cheiloplasty technique contributed to improvements in vertical symmetry and some degree of anteroposterior alignment, with notable correction of vertical upper lip shortening. In contrast, our study employed a modified Tennison cheiloplasty technique, which may partly account for the differing outcomes in anteroposterior symmetry compared to those observed by Al-Rudainy et al. [41] and colleagues. The key difference was the reduced anteroposterior prominence on the cleft side in Al-Rudainy’s study [41], whereas prominence at the point of scar was observed in our sample during the whole study period. This discrepancy may result from underlying genetic growth deficiencies, elevated positioning of the upper lateral nasal muscles, or a combination of these factors.

Our results confirmed that the DA is not only evident in the middle face but also extends to the temporal and buccal regions, especially in children with UCLP. This was in agreement with Moslerová et al. [56], who observed similar trends in a sample of older children (3 to 4.5 years). However, the DA of the frontal region was comparable to healthy controls and therefore it can be concluded that it is probably not related to clefts. Frontal asymmetry is likely a remnant of postural/positional plagiocephaly in infancy, where its prevalence can be as high as 50% in infants [61]. The asymmetric buccal and zygomatic region was typical of patients with UCLP (in infancy and in toddlerhood as well), indicating a lack of growth of this region after surgery, resulting in morphological differences compared to controls [62].

It was confirmed that patients with CL are slightly more symmetrical compared to children with UCLP. The asymmetry slightly progresses with increasing age in the cleft area, and especially in the UCLP group. Overall facial asymmetry is more similar in older children than in younger. Several studies have confirmed that facial asymmetry often progresses slightly with age, especially at the point of cleft in patients with UCLP [41]. On the other hand, the asymmetry deepens in 2-year-olds in our sample, especially in the buccal region and nasolabial region.

### Limitations and future direction of the research

We would like to mention a few limitations of our study and outline potential future directions for our research. The first limitation we acknowledge is the sample size. We are aware that using a larger dataset would be preferable. However, this is a relatively rare sample of children, making it difficult to gather a larger dataset in real time.

Another related limitation is the statistical power of our analysis. Based on a post-hoc power analysis using a permutational multivariate approach (RRPP), we achieved a moderate statistical power of approximately 0.52 (at α = 0.05), which remains below the commonly recommended threshold of 0.8. To attain this recommended statistical power, our analysis suggests that approximately 50 subjects per group would be necessary. However, given the rarity of the studied cleft conditions, recruiting such numbers in practice is challenging. Despite this limitation, the use of robust multivariate statistical methods ensures meaningful interpretation of the observed differences between groups.

Another limitation could be the short period for tracking of facial growth. In this study, we primarily aimed to capture the condition of individuals shortly after birth and in other group shortly after palatoplasty at age two. These two time points are critical milestones in the development of the craniofacial complex, and understanding the processes occurring during these periods is important. Looking ahead, we plan to create the longitudinal dataset of these individuals and also create dataset from a longer-term perspective.

Also from a long-term research perspective, we would like to incorporate more landmarks into the analysis, which could provide more detailed information on left-right asymmetry shifts. This will, however, depend on the facial structure of older children as they grow.

### Clinical implications

Our findings of the facial DA in children with orofacial clefts have several important clinical implications, particularly concerning early intervention strategies in relation to post-surgical outcomes. Facial symmetry in patients with orofacial clefts is affected at all levels by pre-operative measures, timing or surgical technique [63]. Lip repair is associated with better symmetry in facial structures over time, as early repair supports more natural, balanced growth trajectories in the middle face (upper lip, nose, philtrum) [64]. Given that facial asymmetry is closely linked to both functional challenges (such as feeding and speech) and psychosocial outcomes (e.g. self-esteem and social perception, social distress and the risk of social exclusion and disadvantage), early intervention can mitigate these impacts by achieving better baseline symmetry [65, 66].

Despite the importance of the evaluation of facial symmetry as a clinical outcome parameter in cleft care, the assessment of facial aesthetics in patients with clefts based on facial symmetry is not well established [13]. The combined methodology of this study allows clinicians to use our insights to support early intervention as a means to minimize facial asymmetry in the long term. Understanding the typical developmental trajectory of facial asymmetry in children with clefts can guide surgical timing, technique, and postoperative assessments. This is particularly relevant in deciding the timing of secondary interventions or revisions, as achieving close-to-normal symmetry early on may reduce the need for extensive future surgeries [14]. Additionally, 3D stereophotogrammetry and similar imaging technologies provide clinicians with precise tools to quantify facial asymmetry objectively, allowing for better tracking of surgical outcomes and tailored adjustments to treatment protocols as children grow.

Overall, the evidence highlights the value of early, multidisciplinary approaches for improving both aesthetic and functional outcomes in children with orofacial clefts, with implications for long-term growth patterns and quality of life [14, 64]. Further research is recommended to optimize these protocols and confirm the long-term benefits of early neonatal cheiloplasty and palatoplasty in connection with asymmetry of the face.

## Conclusions

In conclusion, this study presents valuable results obtained through a combination of methods evaluating 3D landmarks (crucial facial points) and 3D polygonal meshes (whole facial surface). The 3D landmark method allowed us to assess DA in right-left and cranio-caudal shifts, while polygonal mesh analyses allowed us to describe DA in antero-posterior shifts. These two methods provide a comprehensive description of facial DA in all spatial directions. This study assessed directional asymmetry in the faces of healthy children and those with CL and UCLP. The asymmetry evaluation was conducted during a period representing a critical phase for craniofacial development and growth during early childhood (at 0.2–0.5 years of age and at 1.6-2.0 years of age).

In analyses using the 3D landmark-based method and polygonal mesh analysis, 75% of the variability in facial DA was explained by the first four or five Principal Components.

Our results showed that, at the level of 3D landmarks (representing right-left and cranio-caudal asymmetrical shifts), asymmetry was almost absent in the control group and most pronounced in the UCLP group. At a younger age, asymmetry is most evident in the midline and mouth area. In older children, the midline becomes more symmetrical, but the mouth area remains the same, and the chin shifts to the left.

The difference between CL patients of older age and the controls disappeared. This indicates that the DA pattern becomes more similar to healthy children with age, at least in the group with a milder form of cleft.

At the level of polygonal maps visualising antero-posterior asymmetrical shifts, it was found that in healthy controls, asymmetry is slightly greater in the older group, particularly on the right hemiface, especially in the forehead area. It was also found that DA is very pronounced and protrusive on the cleft side in the philtrum region in both the CL and UCLP groups. This protrusion may result from tissue healing through a tissue scar. Overall, DA is most prominent in the group with the most severe cleft deformity – the UCLP group. In this group, growth insufficiency is also evident in the form of hypoplastic areas of the nasal wing and cheek on the cleft side. This concept is further supported by the midline deviation observed in older children in the group with UCLP, which shifts towards the cleft side. In terms of development at two different times, DA tends to increase in the anteroposterior direction (philtrum and cheeks areas), particularly in the UCLP group. However, overall variability in DA between groups decreases in older children, especially between the control group and the CL group.

One of the primary goals in the multidisciplinary treatment of patients with clefts is to substantially improve facial appearance to minimize the psychological impact of this kind of malformation on the patients’ families. The methodology of facial asymmetry research presented in the study can be used as an objective measure of treatment outcome. It can also help identify different developmental trends in the asymmetry of patients depending on cleft severity. It is necessary to mention the need for further follow-up studies that would confirm the results of this research.

## Data Availability

No datasets were generated or analysed during the current study.
